# A patient-specific computational model of hypoxia-modulated radiation resistance in glioblastoma using ^18^F-FMISO-PET

**DOI:** 10.1098/rsif.2014.1174

**Published:** 2015-02-06

**Authors:** Russell C. Rockne, Andrew D. Trister, Joshua Jacobs, Andrea J. Hawkins-Daarud, Maxwell L. Neal, Kristi Hendrickson, Maciej M. Mrugala, Jason K. Rockhill, Paul Kinahan, Kenneth A. Krohn, Kristin R. Swanson

**Affiliations:** 1 Department of Neurological Surgery, Northwestern University and Feinberg School of Medicine, 676 N Saint Clair Street, Suite 1300, Chicago, IL 60611, USA; 2 Northwestern Brain Tumor Institute, Northwestern University, 675 N Saint Clair Street, Suite 2100, Chicago, IL 60611, USA,; 3 Department of Radiation Oncology, University of Washington, School of Medicine, 1959 NE Pacific Street, Seattle, WA 98195, USA; 4 Department of Pathology, University of Washington, School of Medicine, 1959 NE Pacific Street, Seattle, WA 98195, USA; 5 Department of Neurology, University of Washington, School of Medicine, 1959 NE Pacific Street, Seattle, WA 98195, USA; 6 Department of Radiology, University of Washington, School of Medicine, 1959 NE Pacific Street, Seattle, WA 98195, USA

**Keywords:** hypoxia, mathematical modelling, radiation resistance, glioblastoma, patient-specific

## Abstract

Glioblastoma multiforme (GBM) is a highly invasive primary brain tumour that has poor prognosis despite aggressive treatment. A hallmark of these tumours is diffuse invasion into the surrounding brain, necessitating a multi-modal treatment approach, including surgery, radiation and chemotherapy. We have previously demonstrated the ability of our model to predict radiographic response immediately following radiation therapy in individual GBM patients using a simplified geometry of the brain and theoretical radiation dose. Using only two pre-treatment magnetic resonance imaging scans, we calculate net rates of proliferation and invasion as well as radiation sensitivity for a patient's disease. Here, we present the application of our clinically targeted modelling approach to a single glioblastoma patient as a demonstration of our method. We apply our model in the full three-dimensional architecture of the brain to quantify the effects of regional resistance to radiation owing to hypoxia *in vivo* determined by [^18^F]-fluoromisonidazole positron emission tomography (FMISO-PET) and the patient-specific three-dimensional radiation treatment plan. Incorporation of hypoxia into our model with FMISO-PET increases the model–data agreement by an order of magnitude. This improvement was robust to our definition of hypoxia or the degree of radiation resistance quantified with the FMISO-PET image and our computational model, respectively. This work demonstrates a useful application of patient-specific modelling in personalized medicine and how mathematical modelling has the potential to unify multi-modality imaging and radiation treatment planning.

## 1. Introduction

Glioblastoma multiforme (GBM) is the most aggressive primary brain tumour and accounts for the majority of primary brain tumours [1]. Following diagnosis, GBM is often treated with surgical intervention followed by concurrent radiation and chemotherapy [2]. GBM is characterized by invasive tumour cells that can be found as far away as 4 cm from the tumour mass [3]. This tumour cell invasion is not revealed by magnetic resonance imaging (MRI), the principal means of monitoring GBM progression and response to therapy [4].

### 1.1. Routine magnetic resonance imaging for glioblastoma

Clinically, glioblastoma progression and response to therapy are monitored with MRI [4,5]. For the past two decades, routine clinical imaging protocols for glioblastoma have consisted of T1-weighted and T2-weighted MRI sequences, with the addition of the gadolinium contrast agent on T1 MRI (T1Gd) as well as fluid attenuation inversion recovery (FLAIR) based on the T2 MRI [6]. Regions of hyperintensity on the T1Gd MRI correlate with high tumour cell density and ‘bulk tumour’. In contrast, the T2 and FLAIR images reflect vasogenic oedema typically associated with inflammatory response to infiltrating tumour cells, at a much lower density than found on the T1Gd region.

Glioblastoma is a particularly hypoxic neoplasm and is, in part, histologically defined by the presence of endothelial proliferation, angiogenesis and necrosis. The cascade of events which initiate and propagate angiogenesis may involve both acute and chronic hypoxic events leading to a heterogeneously hypoxic neoplasm [7]. Although the human brain is oxygen rich in its native state, glioblastoma tumour cells consume oxygen through cooption of existing vasculature as well as stimulation of new vasculature [7]. Increased hypoxia is a hallmark of aggressive tumour growth [8–10] and is known to reduce the efficacy of radiation therapy (RT) [11] and is negatively correlated with prognosis, although this is debated [12]. In order to investigate the role of hypoxia in mediating response to RT, we studied an MRI-based patient-specific computational model of tumour growth and response to RT and combined it with hypoxia determined with [^18^F]-fluoromisonidazole (FMISO) positron emission tomography (PET) [13,14].

Our patient-specific computational model quantifies a prediction of response to RT using *in vivo* MRI data. Patient-specific growth rates are quantified by net rates of proliferation and invasion and are calculated using routinely available MRI obtained prior to treatment. Response to RT can be described by the linear-quadratic dose–response model [15]. The motivation for this patient-specific computational modelling is the need for more quantitative and individualized medicine that unifies molecular and anatomical imaging modalities as well as incorporates tumour growth and response rates, which have been shown to vary from patient to patient [16–19]. We present an analysis for one GBM patient with MRI and FMISO-PET prior to RT to document our method and demonstrate an approach to quantifying hypoxia-mediated resistance to RT using patient-specific computational modelling.

## 2. Material and methods

### 2.1. Glioblastoma patient case study

We study a single glioblastoma patient with two MRIs and an FMISO-PET study prior to RT. The patient received the standard-of-care chemo-RT following diagnostic biopsy and was followed serially with MRI throughout the disease course. Tumour size data were recorded prior to and following RT to be compared with predictions of a patient-specific computational model. The model was used to investigate and quantify the role of regional hypoxia in determining MRI-defined response to RT.

### 2.2. Natural history and diagnosis

The subject of this study is a 73-year-old man who provided informed consent to participate in an observational study approved by the local institutional review board (IRB). An MRI obtained at the time of presentation demonstrated a left temporal lobe lesion surrounded by oedema. A needle biopsy via a bur hole procedure was performed of the left temporal lobe lesion. Multiple tissue sections revealed areas of glial neoplasm as well as areas of necrosis with associated pseudo-palisading of neoplastic nuclei. On the basis of these pathological findings, the biopsy was most consistent with WHO grade IV GBM [7].

### 2.3. Imaging data

The patient's diagnostic and pre-operative MRIs were performed 13 days apart. Three days after the biopsy procedure and 2 days prior to the first fraction of RT, the patient underwent an [^18^F]-FMISO-PET study on an IRB-approved research protocol. MRI and PET protocols can be found in the electronic supplementary material. The patient underwent an MRI study 3 days following the completion of RT, and subsequent images were taken at two-month intervals. The MRI and PET images were spatially coaligned to a common coordinate system using a rigid body transformation to the BrainWeb phantom [20] using the PFUS package within the PMOD software [21] and statistical parametric mapping, available through the Matlab software suite [22].

### 2.4. Tumour volume data

Tumour volumes were measured for the T1Gd and T2 sequences for MRI studies (table 1) using a semi-automated threshold-based pixel intensity background subtraction software developed in Matlab. The accuracy and reproducibility of this method is comparable to manual tumour delineation [23]. Specifically, tumour volume *V* (cm^3^) was calculated numerically using the formula
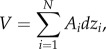
where the image series is composed of *N* two-dimensional slices, acquired in the axial plane, and where slice *i* has thickness *dz_i_* and tumour area *A_i_*. The tumour area *A_i_* is determined by summing the number of pixels containing tumour on slice *i*. The tumour growth velocity, shown in table 1, was calculated based on changes in radius of a sphere of equivalent volume measured prior to biopsy on the T1Gd images.

**Table 1.** MRI and FMISO-PET volumes for the study patient. MRI volumes are taken from the diagnostic image. Velocity of growth was computed from T1Gd MRI volumes.

days between pre-biopsy MRIs	velocity (cm per year)	contrast-enhancing (T1Gd) tumour volume (cm^3^)	T2 MRI tumour volume (cm^3^)	FMISO-PET hypoxic volume (cm^3^)
13	2.43	18.8	44.0	2.4

### 2.5. Quantifying hypoxia *in vivo* with FMISO-PET

**Figure 1.**
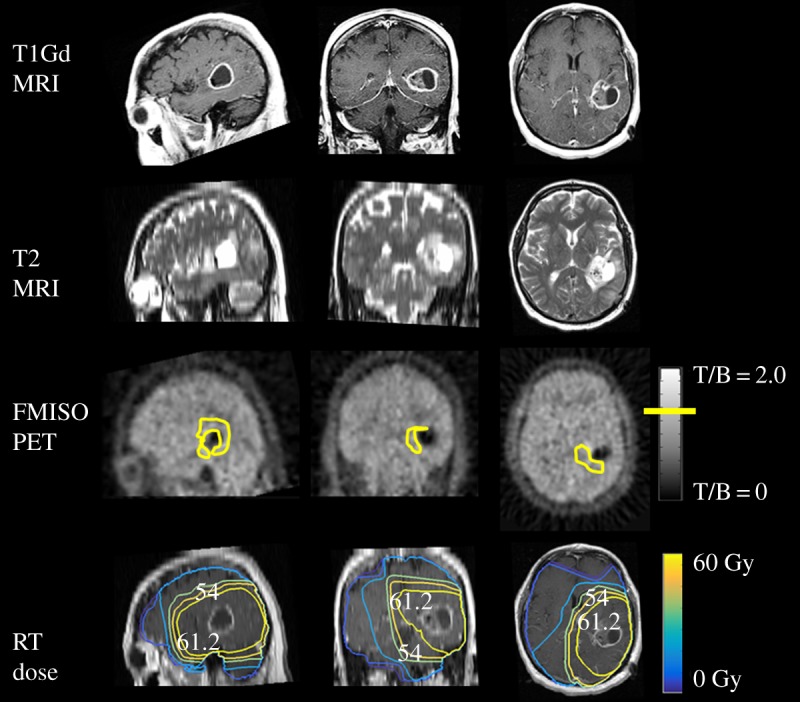
Orthogonal views of the patient's diagnostic T1-weighted gadolinium enhanced (T1Gd), T2-weighted MRI and FMISO-PET obtained prior to RT, with the composite RT dose based on MRI-defined margins. The yellow region of the FMISO-PET image indicates hypoxia as defined by tumour-to-blood values greater than or equal to 1.2 [24]. (Online version in colour.)

The co-registered FMISO-PET images were scaled to the average venous blood concentration of FMISO activity (see the electronic supplementary material for details) to produce a tumour/blood (T/B) ratio image seen in figure 1 [24]. A T/B ratio greater than or equal to 1.2 (T/B ≥ 1.2) was associated with regions of hypoxia and used to determine the total hypoxic volume (HV; table 2) [24]. PET imaging is inherently noisy, and there can be isolated voxels of FMISO uptake scattered throughout the brain; defining HV as T/B ≥ 1.2 and restricting the FMISO signal to the region of T2-weighted MRI abnormality largely excludes this noise and isolates the FMISO signal to the tumour area [25]. The HV is distributed within the tumour, from the bulk tumour mass (T1Gd) to the invasive edge and tumour periphery, defined by the T2/FLAIR abnormality with a 2 cm margin (T2^+^; table 2). The FMISO T/B values in the whole brain have a mean and standard deviation of 0.813 ± 0.223 and are not normally distributed (one-sample Kolmogorov–Smirnov test *p* < 0.0001).

**Table 2.** Hypoxic volume and maximum tumour-to-blood (T/B) pixel value within MRI-defined tumour regions for the patient. T2^+^ is defined to be the T2 MRI abnormality with a uniform 2 cm margin. T2-T1Gd is the T2 region less the contrast-enhancing T1-weighted tumour region, including regions of necrosis. HV is distributed throughout the tumour, from the bulk (T1Gd) to the periphery (T2^+^).

region	HV (cm^3^)	T/B max
T2^+^	2.430	1.523
T2-T1Gd	0.646	1.454
T1Gd	0.698	1.523

### 2.6. Radiation treatment

Five days after the diagnostic biopsy, the patient was treated with RT delivered with a three-dimensional conformal treatment plan using 6 MV photons. The target volumes were defined as the T2-defined abnormality with a 2.5 cm margin, which had a planned target dose of 54 Gy delivered in 30 daily fractions of 1.8 Gy per fraction. An additional dose was delivered to a smaller volume defined by the gadolinium-enhancing region plus a 2 cm margin of 7.2 Gy delivered in an additional four daily fractions of 1.8 Gy per fraction for a total of 61.2 Gy to this region. The target volumes are labelled as isodose curves of 61.2 and 54 Gy on the planning MRI in figure 1. Radiation was delivered with concurrent temozolomide (TMZ) 75 mg m^−2^ given daily during course of RT and continued adjuvantly for a total of 14 months [2].

### 2.7. A mathematical model of glioblastoma growth and response to radiation therapy

2.1
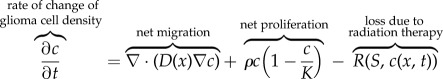
and2.2
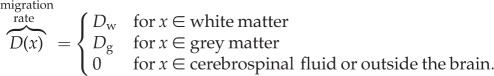
The proliferation–invasion radiation therapy (PIRT) model is a partial differential equation that quantifies the spatial and temporal rates of change of glioblastoma cell density and incorporates the delivery and effect of RT. The model describes tumour cell density, denoted *c* = *c*(**x**, *t*) at time *t* and location **x** = (*x,y,z*) in units cells per mm^3^, in terms of diffuse invasion and density-dependent logistic growth. Logistic growth relates the *per capita* growth rate to available space for the cells to grow, so that if there are few cells in a unit volume of tissue, the overall growth rate is higher than if there are many cells per unit volume. The maximum number of tumour cells that can fit in a cubic millimetre of tissue is known as the carrying capacity, denoted *K,* and is computed to be 1.91 × 10^6^ (cells mm^−3^) assuming a 10 µm diameter tumour cell. Once the tumour cell density reaches the carrying capacity (*c* = *K*) at a particular spatial location, the tumour cells are space-restricted and therefore do not proliferate. Because we do not incorporate the clearance of dead cells, when the tumour cell density reaches the tissue carrying capacity, we assume the tumour cells are dead or become quiescent in this region.

The PIRT model (equations (2.1) and (2.2)) quantifies glioblastoma growth in terms of two net rates: proliferation (*ρ*, per year) and invasion (*D*(**x**), mm^2^ per year). The invasion of malignant tumour cells into the brain parenchyma is influenced by the anatomy of the brain, codified in equation (2.2): tumour cells preferentially migrate along the myelinated axons of neurons composing the white matter and move more randomly and slowly through the dense grey matter which composes the cortical surface and some internal structures of the brain [26,27]. The last term in equation (2.1) represents the loss of tumour cells owing to RT and is based on the linear-quadratic model and the clinical RT plan, discussed below and given in equations (2.3) and (2.4).

### 2.8. Differential motility

**Figure 2.**
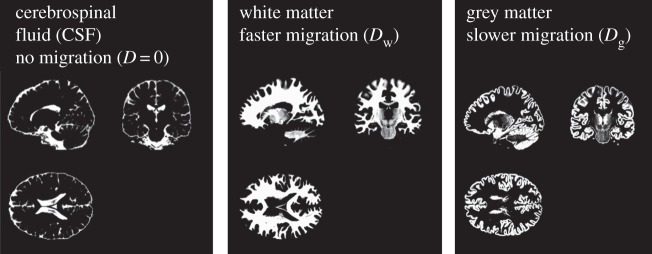
The BrainWeb phantom provides a voxel-wise probability map used to define the invasion rate of the tumour in model simulations [20]. Each voxel is composed of grey matter, white matter and/or CSF in relative proportions such that the sum of all tissues in each voxel is unity. The voxels in the phantom are cubic with dimensions 1 × 1 × 1 mm = 1 mm^3^.

The invasive migration of tumour cells throughout the brain presents a challenge to understanding the true extent of the subclinical disease, as tumour dispersal speeds can vary up to 100-fold between pioneering cells in white matter compared with the more random motion in the core of the tumour and in grey matter [7,27,28]. To model this behaviour, the tumour cell invasion rate (equation (2.2)) is a function of the spatial variable **x**, so that glioma cells migrate 100 times faster in the white matter than in the more dense grey matter, *D*_w_ = 100*D*_g_ [29,30]. The BrainWeb phantom was used for tissue classification, partitioning the brain into grey and white matter in addition to cerebrospinal fluid (CSF) on a 1 mm^3^ cubic grid. The phantom is used to define the invasion rate of tumour cells spatially in the brain *D*(**x**), figure 2 [20,31]. The simulated tumour is initiated as a single voxel of cells located at approximately the centre of mass of the T1Gd-defined tumour volume. The model equations are solved using a numerical approach, with time and spatial grids determined to meet stability requirements (see the electronic supplementary material) [17].

### 2.9. Modelling the effect of radiation therapy on glioblastoma multiforme tumour cell density

2.3
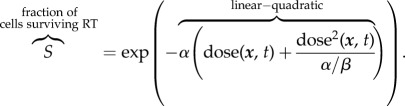
Equation (2.3) is the surviving fraction of cells (*S*) from the linear-quadratic dose–response model. Equation (2.3) converts radiation dose in units of Gy defined in space and time (denoted dose(***x****,t*)) as determined by the patient's clinical radiation treatment plan into a biological effect of each radiation treatment [11,15,32,33]. The linear-quadratic model is the most widely used dose–response model in radiation oncology [11]. First-order repair kinetics are not included in this model because these effects are negligible at dose fractions of less than 2 Gy.2.4
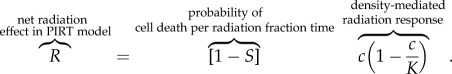


The radiation loss term in the PIRT model (equation (2.1)) is nonlinear and follows the same density-dependent logistic formalism as the tumour growth model, so that the rate of tumour cell death is related to the rate of killing and the density of tumour cells. The probability of cell death (1 − *S*) from the linear-quadratic model (equation (2.3)) determines the rate of cell killing during each treatment fraction time (equation (2.4)). For low cell densities, the effect of RT is linearly related to the fraction of tumour cells killed. This assumption is consistent with the common understanding that cells actively undergoing mitosis are more susceptible to DNA damage and are found at the rim and periphery of the tumour more than the dense core [11]. However, when the cell density is close to the carrying capacity of the tissue *K*, it is assumed that the effect saturates owing to increased interstitial pressure and decreased dose per cell. When the tumour cell density reaches carrying capacity (*c* = *K*), there is no radiation effect. This situation corresponds to an unresponsive necrotic core, where we assume that there is no radiation-induced cell killing, because the cells are already dead. A necrotic core is a histologic and radiographic hallmark of glioblastoma that develops when the tumour growth exceeds the tissue carrying capacity.

### 2.10. Patient-specific radiation sensitivity

In order to quantify radiation sensitivity on a patient-specific basis, we use the linear-quadratic model with the ratio *α*/*β* fixed. This allows us to regard *α* as the single parameter to define radiation sensitivity. For each point in space and time, an effective dose and probability of cell survival can be calculated that corresponds uniquely to the individual patient's treatment plan and the linear-quadratic dose–response model parameter *α*. Increasing *α* decreases the probability of cell survival, *S*, and therefore increases the probability of RT-induced cell death. Increasing values of *α* correspond to increasing treatment effect and deviation from untreated growth. With the ratio *α*/*β* fixed, the single parameter *α* can be uniquely determined using either the T1Gd or T2 post-chemoradiation tumour size, using a bootstrap optimization technique, yielding a one-to-one relationship between *α* and model prediction error, as described in Rockne *et al.* [17]. The first T2 MRI following chemoradiation was used to determine the radiation response parameter *α* owing to the localization of FMISO-PET activity within the T2^+^ region (table 2).

### 2.11. Model of hypoxia-modulated radiation resistance

The ratio of the parameters *α*/*β* in the linear-quadratic model provides a measure of tissue response to radiation exposure. In our treatment simulations, the ratio *α*/*β* is held constant at 10 Gy, which is a reasonable assumption for tumour tissue and consistent with previous work [11,25,34–36]. The relative contribution of the ratio *α*/*β* to radiation response is modulated by the presence of hypoxia. We use the scaling oxygen enhancement ratio (OER), which determines relative resistance to RT in regions of low oxygen [37–39]. The implementation of the OER to the linear-quadratic model is to modify the radiobiological parameters *α* and *β* to be spatially defined, so that the OER is applied in regions of hypoxia, as follows:2.5
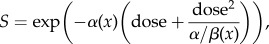
2.6
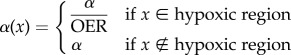
2.7
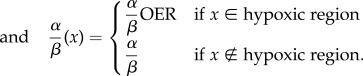
The OER assumes values between one and three [40], corresponding to no hypoxic effects and maximum effect, respectively. Although the OER is often considered a function of oxygen tension measured in units of mm Hg, we associate the T/B FMISO-PET image with a volume fraction of hypoxia and implement the OER in a binary, voxel-wise manner as shown in equations (2.5)–(2.7).

### 2.12. Quantifying hypoxia-mediated radiation resistance with the proliferation–invasion radiation therapy model

The PIRT model is used to investigate the role of FMISO-PET-defined hypoxia in determining model-predicted radiation response and to test the sensitivity of our definition of hypoxia in influencing radiographic response to RT. Each simulation was run using an anatomically accurate three-dimensional brain phantom [20] and the patient's three-dimensional conformal radiation dose prescription extracted from the treatment planning system (Philips Pinnacle). The following treatment scenarios are considered:

(1) Clinical radiation dose delivered with spatially uniform treatment response. This corresponds to a value for the OER equal to one (OER = 1) at all locations in the brain, and assumes that all tumour cells are equally susceptible to RT damage.
(2) Clinical radiation dose with localized radio-resistance owing to hypoxia via a binary relationship. This quantifies focal radio-resistance defined by the patient's FMISO-PET scan, where the tissue to blood value is greater than or equal to 1.2 [24], defines the HV where the OER is modified. Four simulations were performed with the OER equal to 1.5, 2.0, 2.5 and 3.0 to characterize a range of hypoxia-mediated responses. The spatially defined OER is given by2.8
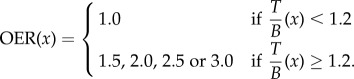

### 2.13. A patient-specific proliferation–invasion radiotherapy model

To establish a histopathological correlation of cellular density to imaging, we associate enhancing features on T1Gd and T2-weighted MRIs with high and low tumour cell densities, respectively [41,42]. One corollary of this estimate is the modelling of a diffuse gradient of tumour cells invisible to imaging. The relative proportion of occult disease is characterized by an invisibility index (the ratio of the model parameters *D*/*ρ* [43]) which has been inversely related to the volume of hypoxia within the tumour measured on FMISO-PET relative to the overall tumour mass on MRI, so that more nodular tumours (small ratio *D*/*ρ*) are more hypoxic than diffuse tumours (large ratio *D*/*ρ*) [9]. This can be interpreted as the difference between vascular cooption and tumour-driven angiogenesis in diffuse versus nodular neoplasms, respectively.

The PIRT model (equation (2.1)) predicts a nearly linear radial growth of the abnormality seen on imaging, which approaches a constant velocity defined by Fisher's approximation 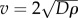 [44,45]. A constant velocity of imageable growth on MRI has been demonstrated for 27 untreated low-grade gliomas [46] computed from serial MRI observations. The invisibility index and velocity of growth are computed from gross tumour volumes and combined to calculate patient-specific net rates of diffusion (*D*) and proliferation (*ρ*) [17,42].

### 2.14. Patient-specific model parameters

Patient-specific model parameters *D* and *ρ* were calculated based on baseline tumour growth kinetics using pre-biopsy MRI. Specifically, within the spectrum of dynamics observed in glioblastoma, the patient's net rates of invasion and proliferation that characterize the tumour growth lie within 1 standard deviation of the population mean observed in 63 patients [16]. Similarly for the patient-specific radio-sensitivity parameter *α*, the calculated value of *α* = 0.055 (per Gy) reflects neither an exceptionally resistant nor sensitive response (table 3). The relative invasiveness or ‘invisibility index’ (*D*/*ρ* = 0.93 mm^2^) reflects a tumour growth pattern which is balanced between cooptive and angiogenic vascularity and therefore predicts a modest hypoxic burden.

**Table 3.** Patient-specific tumour growth and response rates quantified with the patient-specific PIRT model.

net invasion rate *D* (mm^2^ per year)	net proliferation rate *ρ* (per year)	relative invasiveness *D*/*ρ* (mm^2^)	radio-sensitivity *α* (per Gy)
12.84	13.82	0.93	0.055

### 2.15. Spatial metrics of model accuracy

In order to assess the accuracy of model predictions, we compare the simulated and actual tumour regions on T1Gd and T2-weighted MRI using spatially defined similarity metrics. Each metric returns a value indicating the quality of agreement between model and observed tumour growth. This analysis was performed on the two MRIs prior to RT and on the first MRI following RT on both the T1Gd and T2 tumour volumes. Model-predicted RT response is defined with the first MRI performed post-RT [17]. Because we do not explicitly model chemotherapy, we cannot confidently apply this model or analysis to MRI observations beyond the first two post-radiation scans, when adjuvant chemotherapy is often administered. Similarity metrics using algebraic combinations of true-positive (TP), false-positive (FP), false-negative (FN) and true-negative (TN) are computed on a voxel-wise basis and include the positive predictive value = TP/(TP + FP), sensitivity = TP/(TP + FN), specificity = TN/(FP + TN), Jaccard index = TP/(FN + TP + FP) and volume similarity = 1 − (|FP-FN|/(FP + 2TP + FN)). These metrics take values from 0 to 1, with one indicating exact agreement. Model-predicted and observed tumour radii were also compared.

In addition to voxel-wise metrics of concordance, we quantified morphological similarity by computing the distance between the predicted and observed tumour surfaces in three dimensions. This measure returns a distribution of distances, so that a value of zero in this distribution indicates intersection of the simulated and actual tumour surfaces. We report the median and standard deviation of this distribution—the closer to zero and smaller the variance, the better the model prediction. Reporting the median and standard deviation allows us to evaluate both variance and bias in our model predictions and avoid a cancelling effect of including both positive and negative distances that would be reflected in the mean of the distribution.

## 3. Results

**Figure 3.**
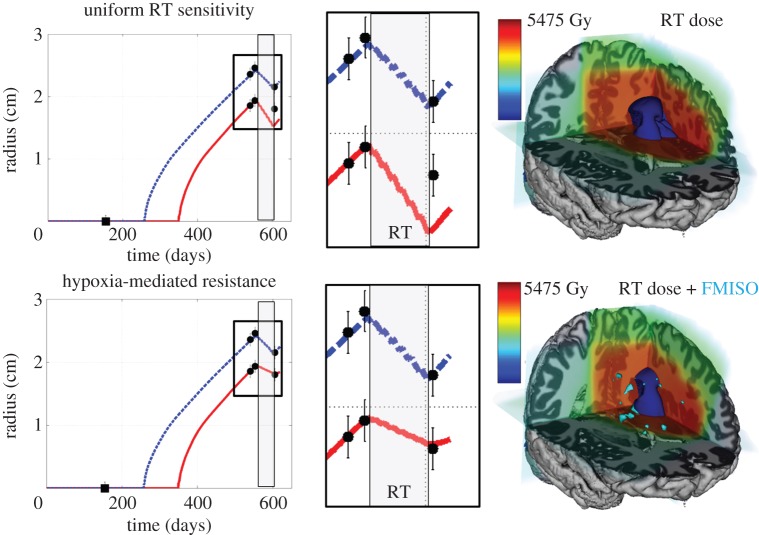
Left column: tumour size versus time. The dashed line is the model-predicted tumour size on T2-weighted MRI, solid line is T1-weighted gadolinium enhanced (T1Gd) MRI. Black circles are tumour sizes calculated volumetrically with 1 mm error bars based on interobserver measurement uncertainty, and the grey rectangle represents when radiation therapy (RT) was delivered. Middle column: zoom-in of tumour size versus time during RT. Right column: three-dimensional renderings of RT dose, FMISO-PET and model-predicted tumour following RT. Top row: patient-specific simulation of RT without the oxygen enhancement ratio (OER) to model uniform sensitivity to RT. Bottom row: simulation with hypoxia-mediated radiation resistance in regions of FMISO-PET T/B activity greater than 1.2.

Using our patient-specific model for glioma growth and response to RT, we find the incorporation of hypoxia-mediated radiation resistance defined with FMISO-PET leads to an order of magnitude decrease in relative volumetric error, from 14.6% to 1.1% (table 4). Incorporation of hypoxia-mediated resistance provided better qualitative and quantitative predictive value to the model in regions of high cellular density where the hypoxia was localized, despite the relatively small volume of hypoxia within the tumour region, representing only 13% of the bulk tumour mass. Relative error on the T2-weighted MRI-based tumour size was improved more modestly, from 0.5% to 0.2%. Absolute differences between volume-based tumour radius ranged from 0.04 to 2.63 mm. Interobserver error in gross tumour radius has been estimated as ±1 mm [47], indicating that simulation predictions are comparable to estimated uncertainty in measurable tumour size, summarized in table 4 and illustrated in figure 3. Additionally, when comparing the PIRT model simulation with and without incorporating the OER, 238% more tumour cells survived owing to local hypoxia resistance effects and 24% of the model-predicted tumour cells prior to treatment were killed by the radiation, indicating a 10-fold overestimate of RT effect without hypoxic resistance.

**Table 4.** Relative and absolute volumetric error between model-predicted post-chemoradiation tumour size and that measured directly from the MRI. Error is reduced by an order of magnitude in the bulk tumour (T1Gd) where the hypoxia is localized.

	MRI region	relative error volumetric radius (%)	absolute error volumetric radius (mm)
Post-RT prediction with focal FMISO-PET radiation resistance	T1Gd	1.10	0.2
	T2	0.20	0.04
Post-RT prediction with uniform radiation sensitivity	T1Gd	14.60	2.63
	T2	0.50	0.11

Spatial concordance between the model-predicted disease burden prior to and following RT was measured through voxel-wise similarity metrics. These metrics are challenged by the spatial alignment of the clinical MRI with the atlas and by a large number of true negatives for pixels far outside the gross tumour region. With this limitation in mind, we observed an increase in voxel-wise concordance by incorporation of localized hypoxia-modulated radiation resistance for most metrics. Volume similarity was increased from 0.844 to 0.91, and the Jaccard metric was increased from 0.72 to 0.83. Sensitivity increased from 99.75 to 99.87, although specificity decreased from 30.25 to 15.61.

**Figure 4.**
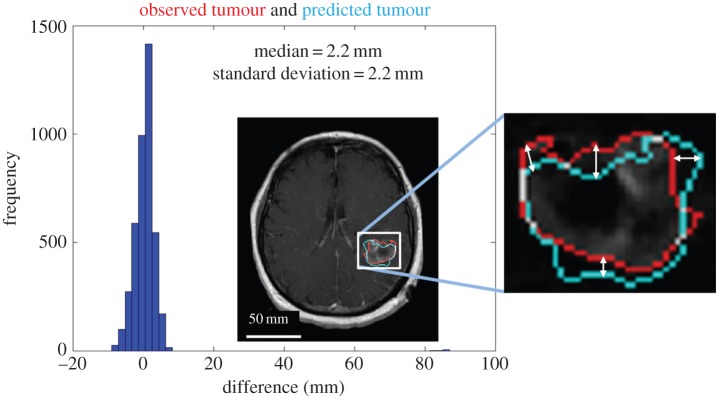
Spatial metric between the model-predicted T1Gd surface (light/cyan contour) and the observed tumour boundary (dark/red contour) on the second pre-RT MRI, indicating a median (±standard deviation) of 2.2 ± 2.2 mm using the observed tumour region (dark/red) as ‘true’. Similar accuracy was observed for the post-RT MRI (table 4). Negative distances indicate an under-estimation of the model-predicted tumour front, whereas positive distances indicate an over-estimation, with zero distance indicating intersection of the surfaces. (Online version in colour.)

The distance between surfaces analysis was performed for all three MRI studies, two prior to biopsy and one immediately following the full course of RT on T1Gd and T2 sequences are illustrated in figure 4. The median distance (±standard deviation) between measured and predicted surfaces, not including overlapping voxels with a distance measure of zero, decreased on T1Gd from 2.8 ± 2.0 to 2.2 ± 2.2 mm and were unchanged on T2, from 2.8 (±3.4). These results indicate an improved model–data agreement in the tumour core (T1Gd) where the hypoxia is localized and a stable model prediction in the tumour periphery (T2), with an overall bias of overestimating the outward growth of the tumour.

### 3.1. Sensitivity analysis

To investigate the sensitivity of our predictions to definitions of hypoxic regions and parameter selection, we considered a range of OER values in the HV ranging from 1.5 to 3.0. This produced a parabolic relationship between relative volumetric error and OER using T1Gd size such that the minimum model–data error occurred at approximately OER = 2.5. OER values between 2.0 and 3.0 yielded relative volumetric errors at or below approximately 1%, and all resulted in an order of magnitude improvement to the model prediction. This analysis demonstrates the robustness of the model predictions to variations in OER, and by extension, the volume fraction of hypoxic cells the T/B FMISO-PET image represents.

To further investigate the role of spatial localization of radiation resistance, simulations were also performed in isotropic three-dimensional spherical symmetry without anatomical complexity, using the T1Gd isodensity as a lower bound threshold to determine the hypoxic region. In these simulations, we observed a similar outcome, that is, that model–data agreement is substantially improved with the inclusion of hypoxia-mediated radiation resistance, where HV is within the bulk tumour region. However, the model predictions were not as accurate with this simplification. This suggests that the contrast-enhancing region is not a suitable surrogate for hypoxic tumour, and that a more specific indicator such as FMISO-PET in three dimensions not only improves the model prediction, but is also needed in a spatially heterogeneous tumour such as GBM. Sensitivity of model-predicted tumour burden following RT based on variability in the tumour growth (*D*, *ρ*) or response rates (*α*, *α*/*β*) is discussed in detail in the supplement to Rockne *et al*. [17] and is not sufficient to account for the improvement in model–data agreement.

### 3.2. Limitations and considerations

This study focuses on one patient to document our method and demonstrate an approach to quantifying hypoxia-mediated resistance to RT using patient-specific computational modelling. More patients must be studied in order to support our findings that incorporation of FMISO-PET-defined hypoxia improves the predictions of the computational model.

As in our previous work [17], we assume the delivery and effect of RT to be an instantaneous, deterministic event using the linear-quadratic equation and its corresponding probability of cell survival/death. Concurrent chemotherapy is assumed to be included in the net effect of RT and is not modelled explicitly. The survival benefit of adding TMZ chemotherapy to radiation treatment was demonstrated in the landmark study by Stupp *et al*. [2] and established a ubiquitous standard of care for newly diagnosed glioblastoma. However, it remains an open question of how best to translate the additive therapeutic benefit of TMZ into a mathematical model. The assumption that the effect of TMZ is included in the response rate parameter *α* does restrict the interpretation of the model predictions for this study.

To further study the additive effect of TMZ to radiation response, one could identify patient cohorts that received RT alone and compare the radiation sensitivity parameters in that cohort with those who received both RT and TMZ. A paired analysis could be performed to control for variations in the tumour growth rates *D* and *ρ*, as is done in the recent study by Adair *et al.* [48]. For intravenously delivered therapy such as TMZ, the blood–brain barrier will result in heterogeneous drug delivery spatially within the tumour. Advanced imaging such as dynamic contrast enhanced or perfusion MRI could be used to infer a drug concentration gradient within the tumour, and a similar mathematical formalism for the loss of tumour cells owing to RT could be used to model the effects of TMZ. A study of this kind would aid in the development of a TMZ model that could be used to study the effects of TMZ that is administered alone following chemoradiation.

The patient's stereotactic needle biopsy was not modelled, as the volume of tissue removed was at the core of the tumour and not a significant portion of the overall tumour mass. The inflammatory response of the tumour and normal appearing surrounding brain owing to surgery may impact MRI abnormalities, particularly on T2/FLAIR. We assume any such effects arising from only a needle biopsy did not impact response to RT, presentation of disease on follow-up imaging or tumour progression. Most glioblastoma patients receive extensive surgical removal of their lesion, which can be modelled by setting that portion of the computational domain and tumour model to zero.

This study assumes no changes to hypoxia through the course of radiotherapy, which does not include the effects of re-oxygenation that could change the distribution of hypoxia within the tumour [49]. The linear-quadratic model does not reflect myriad repair processes and micro-environmental changes induced by radiotherapy [50], although extensions to the L–Q model exist to approximate the effects of re-oxygenation and hypoxia [51]. Hypoxia dynamics can be studied with kinetic FMISO-PET imaging [52]; however, this approach remains relegated to the imaging time point. Moreover, we consider hypoxia a binary variable, which does not account for intravoxel heterogeneity. A more detailed mathematical model of the angiogenic process as it relates to hypoxia could be implemented to explore this heterogeneity, as proposed in [53,54].

Finally, this approach is driven by imaging modalities and does not account for molecular or genetic heterogeneity that is known to exist within glioblastoma, based upon data generated by the TCGA Research Network: http://cancergenome.nih.gov/ [55]. It has been shown that the molecular subtypes defined by the TCGA vary spatially within a single patient's tumour [56], suggesting that a single molecular subtype may not be sufficient to characterize a patient's disease. This emphasizes the need for targeted and image-localized biopsies within various regions of the tumour, as shown by Gill *et al.* [57]. To account for genetic and molecular heterogeneity influence in imageable response to treatment, one would need a multi-scale, patient-specific model framework. Such an undertaking is not attempted here, although the authors recognize that molecular alterations likely play an important role in determining tumour evolution and response to treatment.

## 4. Discussion

We have investigated a computational model of human glioblastoma growth that incorporates hypoxia-mediated resistance to RT based on FMISO-PET in the complex architecture of the brain on a patient-specific basis. Incorporation of focal radiation resistance improves model–data agreement, measured with a variety of metrics, despite the small hypoxic burden (13%) of the tumour. The model predictions are improved by the incorporation of an OER to approximate the degree of radio-resistance created by hypoxic conditions within the tumour. The large improvements in model–data agreement attributable to a modest volume of hypoxia-mediated resistance to radiation effect underscores the significance of spatial heterogeneity in delivery and response to RT in glioblastoma and the complexity of a three-dimensional model.

Without the incorporation of OER and localized radiation resistance, the model is unable to fully capture post-RT tumour size in regions of high cellular density, motivating the addition of the OER parameter derived from *in vivo* clinical data. Ideally, a more patient-specific, biologically driven model of the process of angiogenesis, hypoxia and necrosis using a modelling approach which integrates multi-modality imaging is desired [53,54]. In conjunction, prospective interventional imaging studies which capture changes in intratumoural hypoxia throughout the course of therapy are needed in order to improve models of hypoxia-driven resistance to RT to account for changes in the hypoxic state of the tumour. Moreover, daily fraction radiotherapy likely introduces phenotypic and genotypic selection pressures which eliminate the sensitive tumour cells and leave the most aggressive, resistant clones to repopulate following therapy completion [58].

The goal of modelling biological response to therapy has been long sought after, but there have been no demonstrable successes with the potential for impacting individual treatment planning [59]. Other efforts to incorporate biological effect into radiation treatment plans have either relied on static features of the tumour [60] or are not capable of being truly patient-specific because of the large number of parameters to be estimated [61–65]. We have developed a technique to incorporate proliferation, invasion and response to RT of the tumour over the time course of treatment within the three-dimensional anatomy of the brain. Granting all the assumptions and limitations of the simple proliferation–invasion tumour growth model, this framework has provided a methodology to investigate the role of FMISO-PET-defined hypoxia in modulating radiation response *in vivo* quantitatively. By providing quantitative metrics of a patient's response to radiotherapy, our model has the potential to unify multi-modality imaging and treatment planning and establish a useful application of patient-specific modelling in personalized medicine.
